# The potential of liquid biopsies for the early detection of cancer

**DOI:** 10.1038/s41698-017-0039-5

**Published:** 2017-10-17

**Authors:** Ellen Heitzer, Samantha Perakis, Jochen B. Geigl, Michael R. Speicher

**Affiliations:** 1 Institute of Human Genetics, Medical University of Graz, Neue Stiftingtalstraße 6, A-8010 Graz, Austria; 2 BioTechMed-Graz, Graz, Austria

## Abstract

Precision medicine refers to the choosing of targeted therapies based on genetic data. Due to the increasing availability of data from large-scale tumor genome sequencing projects, genome-driven oncology may have enormous potential to change the clinical management of patients with cancer. To this end, components of tumors, which are shed into the circulation, i.e., circulating tumor cells (CTCs), circulating tumor DNA (ctDNA), or extracellular vesicles, are increasingly being used for monitoring tumor genomes. A growing number of publications have documented that these “liquid biopsies” are informative regarding response to given therapies, are capable of detecting relapse with lead time compared to standard measures, and reveal mechanisms of resistance. However, the majority of published studies relate to advanced tumor stages and the use of liquid biopsies for detection of very early malignant disease stages is less well documented. In early disease stages, strategies for analysis are in principle relatively similar to advanced stages. However, at these early stages, several factors pose particular difficulties and challenges, including the lower frequency and volume of aberrations, potentially confounding phenomena such as clonal expansions of non-tumorous tissues or the accumulation of cancer-associated mutations with age, and the incomplete insight into driver alterations. Here we discuss biology, technical complexities and clinical significance for early cancer detection and their impact on precision oncology.

## Introduction

Precision medicine is defined as the selection of targeted therapies based on an improved understanding of the genetic basis of disease.^1^ Due to the increasing feasibility of sequencing tumor genomes at affordable costs, genome-driven oncology appears to be within grasp to improve the clinical management of patients with cancer.^2,3^ However, at present precision oncology has yet to prove that it can fulfill its promises and produce long-lasting remission and extend survival.^4,5^ Challenges include the enormous biological and clinical complexity of cancer, which is further complicated by the significant intratumor heterogeneity,^6^ and the impact of the tumor microenvironment.^7^ Furthermore, genomes of cancer cells are unstable and may frequently acquire novel changes.^8^

“Liquid biopsies” are based on the analysis of circulating tumor cells (CTCs), circulating tumor DNA (ctDNA), or tumor-derived extracellular vesicles, which have been shed from tumors and their metastatic sites into the blood. Multiple studies have described how molecular information about parent tumors can be extracted from liquid biopsies and a number of comprehensive reviews were recently published on CTCs,^9–15^ ctDNA,^9,12,13,16–23^ and exosomes or extracellular vesicles,^17,24–26^ respectively.

Our own previous work has focused on the assessment and evaluation of somatic copy number changes as well as mutations from CTCs and ctDNA^19,27–33^ and has mostly been done—as the majority of other studies—at advanced disease stages. However, there are increasing efforts by us and many others to move to early disease stages. Perhaps the most ambitious efforts to this end are being conducted by GRAIL, a company that describes its mission as “detecting cancer early, when it can be cured” (https://grail.com). This ambitious aim is supposed to be accomplished by strategies including ultra-broad and ultra-deep sequencing, bioinformatics, and large population-based clinical studies.^34^

Indeed, as cancer is caused by a sequential series of alterations in specific cancer genes that affect the function of certain pathways and usually takes several decades to develop, the vast majority of cancers are not detected in the first 90% of the cancers’ lifetimes.^35^ Due to the increasing knowledge about the driver genes involved and the pathways causing cancer, the question arises whether liquid biopsies may enable novel strategies for early diagnosis. We will focus in this review on this question and to this end we discuss the appertaining biological and technical issues related to identifying small alterations in a complex biological structure such as the human body.

## Plasma DNA diagnostics in physiologic and pathologic conditions

**Fig. 1:**
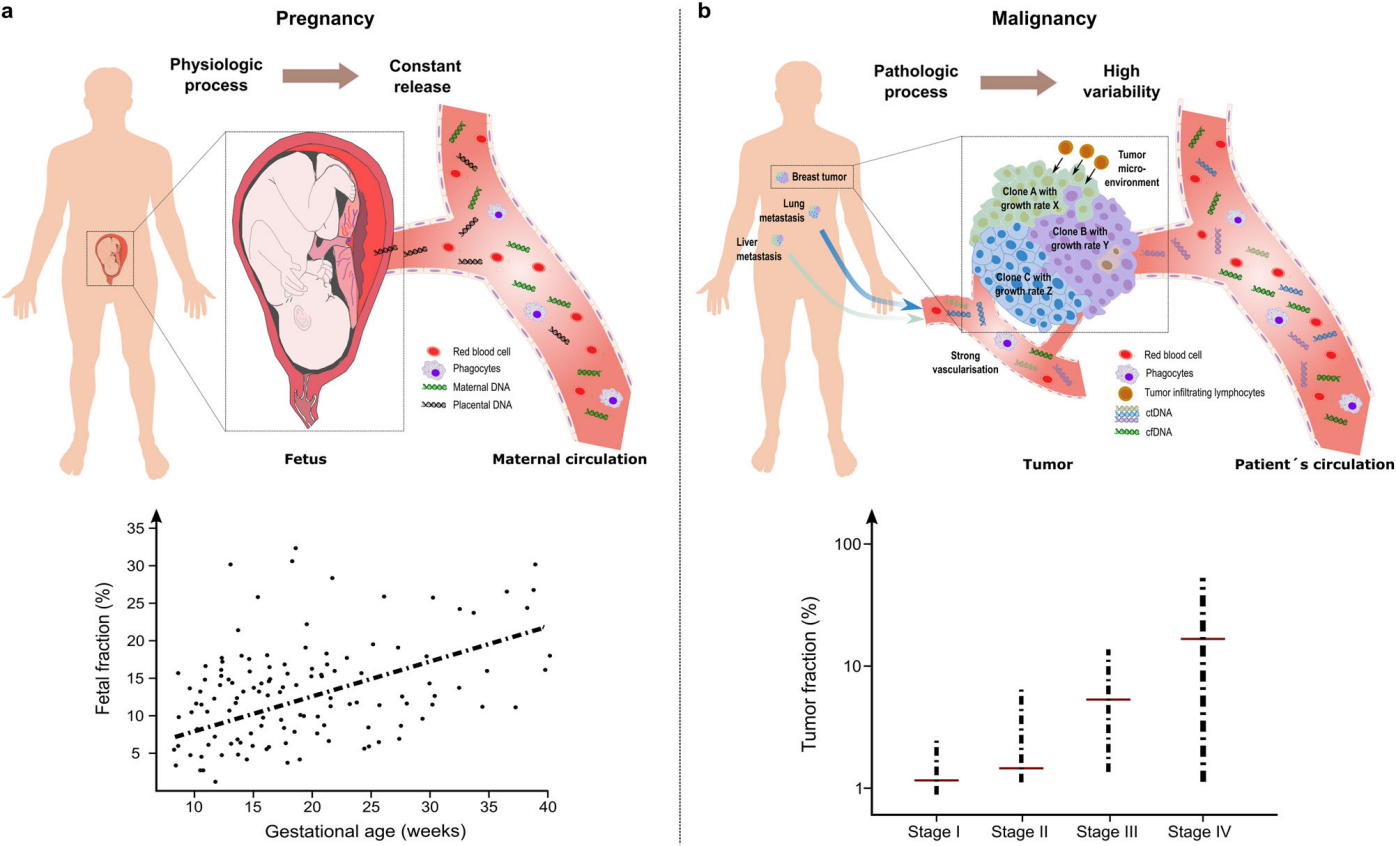
Plasma DNA diagnostics in physiologic and pathologic conditions. **a** Pregnancy is a physiologic scenario which results in a relatively constant and reproducible, i.e., similar in different pregnancies, release of fetal or placental DNA into the circulation. Hence, diagnostic procedures are easy to standardize. The graph at the bottom indicates the fetal plasma DNA fraction as a function of gestational age and shows a positive correlation. In the majority of pregnancies fetal fractions of more than 4%, which is considered to represent a threshold for reliable non-invasive prenatal testing, are already present at 10^th^ week of pregnancy (graph adapted from ref. 126). **b** In contrast, cancer is a pathologic process, which is often heterogeneous (various clones within the primary tumor are depicted in different colors and furthermore metastatic sites, which also contribute) including multiple parameters, e.g., the microenvironment (indicated here by tumor infiltrating lymphocytes) and access to blood vessels, which affect the release of tumor DNA and which may cause significant variation from one patient to the next. At the bottom, average ctDNA levels for tumor stages I to IV are depicted. However, as indicated by the bars, these values may vary tremendously for each stage (graph adapted from ref. 42) and are frequently below 4% required for NIPT. For clarity, we only show DNA fragments in the blood vessels, although other factors, e.g., extracellular vesicles, or modifications of the DNA either by epigenetic changes or alterations in the nucleic acid sequence can also be detected in the systematic circulation

The first description of tumor DNA in the circulation of patients with cancer^36^ preceded the first report about fetal DNA in blood of pregnant females^37^ by several years. However, reliable diagnostic tests were first established for circulating fetal DNA^38^ and testing for fetal aneuploidies from blood of pregnant females, referred to as non-invasive prenatal testing (NIPT), rapidly evolved to a frequently used test capable of detecting trisomies for chromosomes 13, 18, and 21 with high specificity and sensitivity.^39^ In contrast, the development of liquid biopsies for applications in patients with cancer has been much more arduous and several reasons account for this. Pregnancy is a highly reproducible physiological process with minor variabilities and starting from week 9 of pregnancy, the percentage of fetal DNA in the mother’s circulation is relatively high at 10%^37,40^ (Fig. 1a). In fact, with current NIPT technologies a fetal DNA concentration below 4% in maternal plasma is already considered to be prone to result in false negative results.^41^ In contrast, cancer is a pathologic process and a complex, heterogeneous and dynamic disease involving multiple gene-environment interactions affecting numerous biological pathways with multiple variables, such as tumor entity, disease stage or tumor burden, microenvironment, multiple unknown determinants of ctDNA release, and many more (Fig. 1b). As a consequence, the number of mutant DNA fragments can considerably vary and are frequently below 4%, even among patients with the same disease stage.^42^ Hence, ctDNA analysis is much more difficult to standardize, in particular for the detection of early disease stages as outlined in the following.

## Is early tumor detection beneficial at all?

A current belief is that early detection of cancer saves lives and that the earlier a tumor is diagnosed, the better the chance of survival. As this belief was not even shattered by reports that early screening did not prove to be a lifesaver in tumor entities such as those originating from thyroid, prostate and breast, *Nature* listed in a recent editorial “Screening saves lives for all types of cancer” that this is one of the science myths that will not die.^43^

**Fig. 2:**
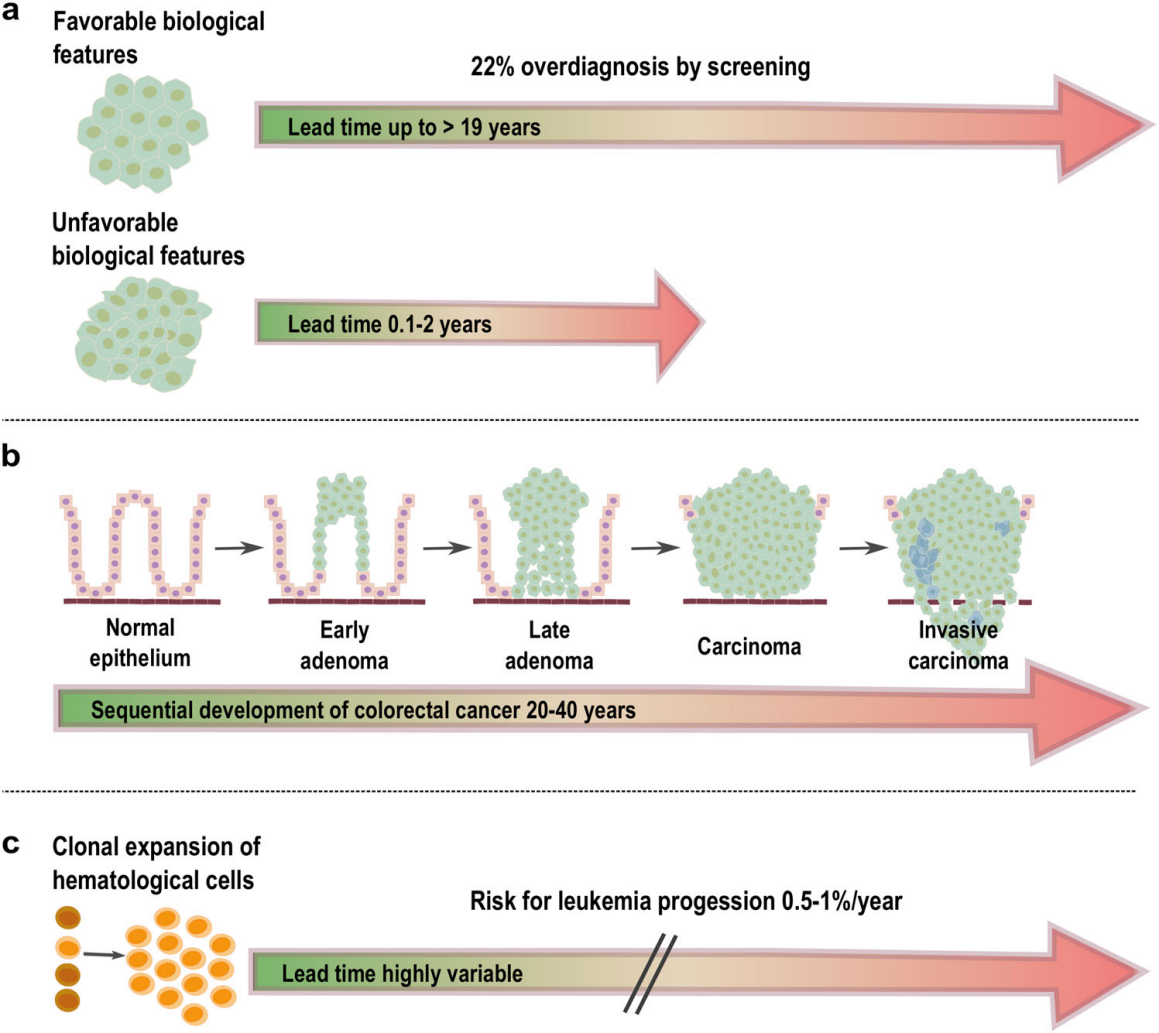
Tumors, clonal expansions and their respective lead times. **a** In breast cancer, tumors with “favorable” biological features (grade 1) may have extensive lead times of up to 19 years and these tumors contribute to significant overdiagnosis by screening mammography. Even if detected at a late stage, these tumors often have an excellent prognosis. In contrast, breast cancers with unfavorable biological features (grade 2–3) usually have short lead times (<2 years) and are therefore less frequently identified by screening mammography. However, because of their biology, early diagnosis would be mandatory to significantly reduce mortality. **b** In CRC, tumors develop through well-defined stages (i.e., stages I–IV), a process which may take up to 20–40 years and is the result of the accrual of specific mutations in tumor driver genes^127^ (image adapted from refs. 127,128). As survival rates are stage-dependent, the earlier the diagnosis is made the better. In the two scenarios depicted in **a** and **b**, the primary clinical challenge remains to determine the fate of the specific lesions so that they do not always differ fundamentally, but transitions exist. **c** Clonal expansions are best characterized in hematopoietic systems and are frequently associated with known driver gene mutations. Their lead time is hard to determine. For CHIP (clonal hematopoiesis of indetermined potential), the odds of progression to overt neoplasia were estimated to be approximately 0.5–1% per year^65^

We discuss the different facets of screening with breast and colon cancer as examples. A recent breast cancer study on screening mammography conducted among women 40 years of age or older revealed a large increase in the incidence of small tumors (<2 cm) and a modest decrease in the incidence of large tumors (≥2 cm).^44^ However, small cancers with favorable molecular features (i.e., grade 1) may have lead times, i.e., the length of time between when a cancer can be detected by screening and when it would have become clinically apparent without screening, of more than 19 years^45^ and are therefore often not destined to progress to large tumors within the lifetime of the patient (Fig. 2a). These tumors contribute substantially to the overall rate of overdiagnosis by screening, which was estimated to be as high as 22%.^44^ Furthermore, these tumors still have an excellent prognosis after progression and can be treated effectively at clinical presentation, thereby offering little benefit to detecting them early.

In contrast, the prognosis for tumors with unfavorable biologic features (i.e., grade 2–3) is considerably better if they can be diagnosed when under 2 cm in size. Unfortunately, because of their short lead times (<0.1–2.0 years^45^), they are rarely diagnosed early and therefore are substantially underrepresented among small tumors (Fig. 2a). Overall, the reduction in breast cancer mortality after the implementation of screening mammography was not attributed to screening but to improved systemic therapy.^44^ Of note, there are exceptions, as breast cancer screening for carriers of *BRCA1* and *BRCA2* germline mutations is recommended and surveillance programs with proven efficiency exist.^46^

However, screening is clearly associated with a reduction in colorectal cancer (CRC) mortality. In the US, a nearly 50% decrease in CRC incidence and mortality is attributed to screening^47^ and, vice versa, 63% of CRC deaths may be due to lack of screening.^48^ The importance of screening and early detection is also reflected in the stage-dependent survival rates in CRCs, which are 94, 82, 67, and 11% for stages I, II, III, and IV, respectively^49^ (Fig. 2b).

Important biological differences between breast and colon cancer may explain the disparities in screening efficiency. There is evidence that low-grade and high-grade breast cancers arise by different molecular mechanisms and it is very rare for a low-grade tumor to dedifferentiate into a high-grade tumor.^50^ In contrast, CRC begins with the growth of adenomatous or sessile serrated polyps and the majority of polyps become dysplastic through one of two major pathways, i.e., chromosomal or microsatellite instability, and subsequently develops into malignancy so that virtually all stage IV colon cancers start out as stage I cancers^51^ (Fig. 2b).

Hence, screening may be particularly efficient in cancer types with well-defined precursor stages that would transform with a high likelihood into malignant disease. In CRC, a liquid biopsy early detection screening test would have to compete and eventually outperform established screening methods, such as guaiac-based fecal occult blood testing, fecal immunochemical test, sigmoidoscopy or colonoscopy.^52^ In breast cancer, such a liquid biopsy test would have to be informative regarding the biology of a lesion, i.e., favorable or not, in order to reduce the aforementioned rate of overdiagnosis and would then offer advantages compared to screening mammography. However, despite our knowledge about the probabilistic fate of a population of lesions observed in specific patient cohorts, it remains important for individual risk estimates to determine the biology of lesions in the context of competing causes of morbidity or mortality in the respective patient. This will be of importance in light of the growing recognition that “clonal expansions” of cells are not a rare phenomenon as outlined in the next section.

## Mosaicism and clonal expansion phenomena

Somatic mutations increase with age and accrue at higher rates in fast renewing tissues.^53^ Mutations in adult stem cells have an especially large impact on the mutational load of tissues because of their potential for self-renewal and capacity to propagate mutations to their daughter cells.^54,55^ In fact, using cells capable of forming long-term organoid cultures to determine genome-wide mutation patterns in single adult stem cells from the small intestine, colon and liver, a high mutation rate of around 36 mutations per year was found.^56^ Based on these data, it is not surprising that an extensive analysis of 140 benign tissue samples representing nine solid tissues (bladder, breast, head and neck, liver, lung, prostate, stomach and thyroid), and corresponding blood revealed multiple exonic mutations in 80% of these samples.^57^ Importantly, the cell of origin analysis indicated that many of the mutations detectable at tissue-level resolution were acquired in the long-lived tissue stem or progenitor cells.^57^

If a mutation occurs in a driver gene, it can confer a fitness advantage which allows this cell to expand and to form a group of identical daughter cells.^58^ Ample evidence for the existence of such clonal mosaicism has been reported.^59–61^

More recently, clonal expansions due to a mutation in a driver gene, which may be also associated with myelodysplasia or leukemia, such as *DNMT3A*, *TET2*, or *ASXL1*, and which enable a hematopoietic stem cell to expand clonally, have been extensively documented.^62–64^ As a consequence, these somatic mutations become detectable in blood-derived DNA with different variant allelic frequencies (VAFs).^62–64^ These VAFs may range from 0.008 to 0.1, which corresponds to 1.6–20% of nucleated cells in circulating blood being derived from mutant hematopoietic stem cells.^62–64^ These observations resulted in the definition of a clonal hematopoiesis of indetermined potential, which refers to the detection of at least one driver mutation in one of the aforementioned genes with a VAF ≥ 0.02 without overt hematologic disease, and represents a pre-cancerous condition with a risk of progressing to leukemia of 0.5–1% per year^65^ (Fig. 2c).

A recent study refined the prevalence of clonal hematopoiesis, as driver mutations were observed with exponential increase with age (20–29 years: 2.5%; 30–39 years: 3.2%; 40–49 years: 8.2%; 50–59 years: 13.2%; 60–69 years: 20.6%).^66^ The increase in prevalence and number of driver mutations was not linear, suggesting that age associated factors may accelerate the occurrence of driver mutations over time.^66^

For solid organs, evidence for such clonal expansion is more difficult to establish as appropriate tissue is more difficult to assess, but evidence for clonally expanded cell populations has been reported for skin,^67,68^ ovarian cells,^69^ and brain.^70^ Such clonal expansions of apparently benign tissues may also be associated with cancer gene somatic mutations as, for example, recently shown in endometriosis.^71^ This occurrence of cancer-associated mutations in benign tissue can pose challenges for early detection of malignancies using liquid biopsy. This was shown in a recent study which used an assay specifically designed to accurately detect *TP53* mutations at very low allelic fractions, in which cfDNA *TP53*-mutated fragments were found in 11.4% of 123 matched non-cancer controls.^72^

## The three early cancer detection scenarios

**Fig. 3:**
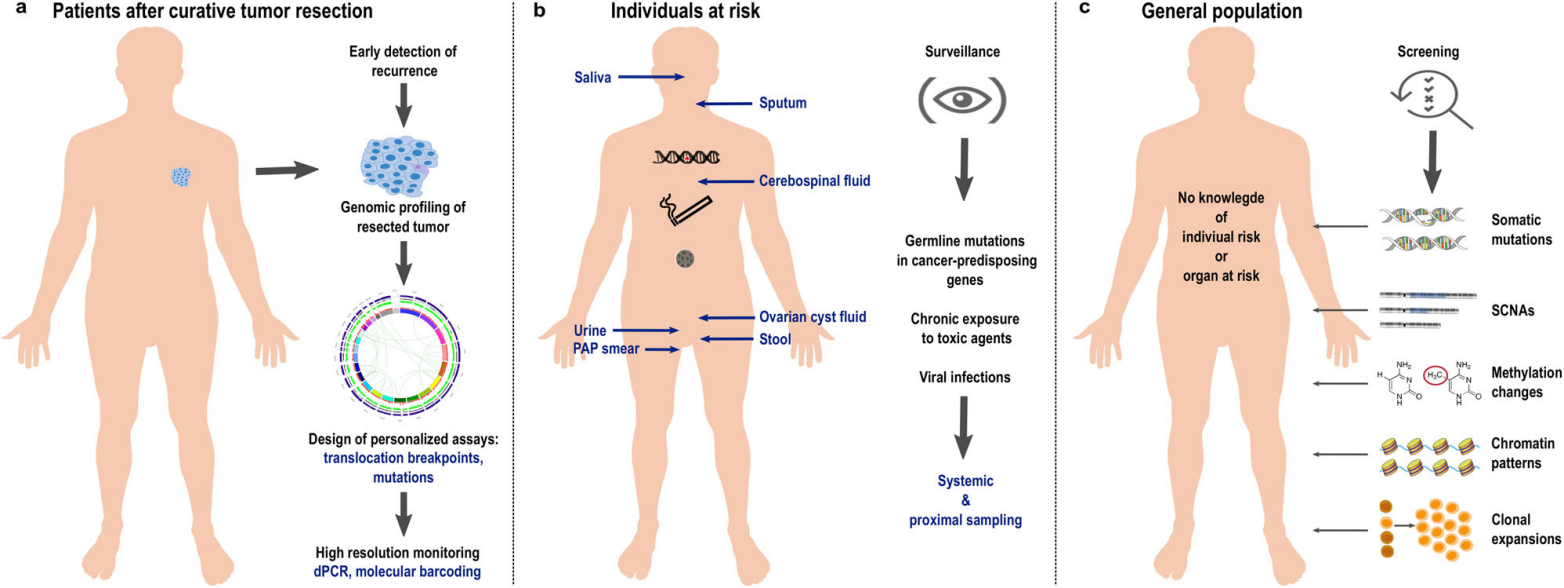
The three early cancer detection scenarios. **a** The detection of relapse after surgery with curative intent is facilitated by the option to profile the resected tumor and to use this information for the design of personalized assay panels, which can be used for high-resolution monitoring approaches. **b** In individuals at-risk, i.e., due to a cancer-predisposition germline mutation, chronic exposure to toxic agents, or due to viral infections, systemic screening approaches can be extended by proximal sampling, i.e., the analysis of other body fluids than blood which are close to the organ with high-risk of malignant transformation. **c** In the “general population”, i.e., persons without a family history of cancer or known risks for tumors at certain sites, liquid biopsy concepts for screening may include the search for mutations, somatic copy number alterations, or analyses of methylation and chromatin patterns. However, generally accepted strategies do not yet exist. Naturally occurring phenomena such as the aging associated mutation rate or clonal expansions of non-tumorous tissue may hamper early detection efforts (see also Table 1)

Recent studies suggested that millions of cells are needed in order for mutations to become detectable in the peripheral blood.^73–75^ Even then, detectable somatic mutations often have low VAFs, but appropriate technologies employing massively parallel sequencing technologies, molecular barcodes, and sophisticated bioinformatics strategies enable the detection of such rare DNA fragments in the circulation^76–80^ with VAFs as low as 0.0025% (2.5 in 10^5^ molecules).^81^ With these requirements in mind, we next address the question whether early detection of cancer by liquid biopsy may be possible and to this end we distinguish three scenarios (Fig. 3). Furthermore, we summarize specific distinctions and challenges of liquid biopsy technologies whether they are applied to pre-malignant lesions and earlier neoplastic stages versus advanced cancers in Table 1.

**Table 1:** Biological and technical differences for applying liquid biopsy technologies on precancers and earlier stages of neoplastic development versus advanced cancers

Parameter	Precancers/early stages	Advanced cancers
Size of lesion	Usually small ( < 1 cm^3^)	Large (≥1 cm^3^)
Clinical signs	Usually none	Apparent
Detectable by imaging	Often not detectable	Yes
Biology of lesion	May range from favorable to unfavorable (refs. 44, 45)	Advanced cancers have in general unfavorable (sub)clones (ref. 6)
Presence of established other tumor markers (e.g., PSA, CEA, CA 125)	Uncertain (ref. 129)	Frequently available, but without high specificity/sensitivity; useful for disease monitoring (ref. 129)
Knowledge of genes to be targeted in liquid biopsy assays	Often unknown (refs. 78, 80)	Usually known or can be established from available tumor tissue (refs. 3, 35, 73)
Established driver genes	Often unknown (refs. 130, 131)	Usually known (refs. 3, 35)
Release of tumor DNA into the circulation	Uncertain (refs. 42, 80)	At stage III and IV disease close to 100% of patients (ref. 42)
Applicable plasma DNA technologies	Usually focused high-sensitivity assays (refs. 16–18)	Broad range of targeted and untargeted approaches (refs. 16–18)
Option of proximal sampling	Only if endangered tissue is known (refs. 16, 17)	In selected tumor entities, but frequently not necessary
Option to design personalized assays	Possible, provided that tissue is available (refs. 73, 84, 85)	Tissue is usually available, can be designed for truncal and branch mutations (refs. 73)
Expected VAF of somatic mutations in blood	Extremely low, if present at all (refs. 42, 80)	Frequently high (refs. 32, 42, 107, 132)
Tumor heterogeneity	Relatively low (refs. 6, 133)	High (refs. 6, 133)
Presence of potentially confounding mutations	In particular, persons with increased age may have acquired cancer-associated mutations without ever developing cancer (refs. 65, 71, 72)	Distinction between driver and passenger mutations needed for disease monitoring (ref. 35)
Presence of potentially confounding clones	Clonal expansion of non-tumorous tissue may mimic a malignant event (refs. 65, 67–70)	Likely that all metastatic sites are reflected in plasma DNA analysis (ref. 134)
Detection of SCNAs	Hard to detect due to low VAF at this disease stage (refs. 23, 27)	Often informative and may indicate evolution of novel clones (ref. 32)
Availability of established clinical guidelines	None	Emerging, e.g., EGFR mutation testing as blood-based companion diagnostic for patients with NSCLC

*PSA* prostate-specific antigen, *CEA* carcinoembryonic antigen, *CA 125* cancer antigen 125, *VAF* variant allelic frequency, *SCNAs* somatic copy number alterations, *NSCLC* non-small-cell lung cancer

The first scenario relates to the detection of relapse or recurrence after surgery with curative intent (Fig. 3a). Here, the options for ctDNA profiling to detect relapse are excellent for two reasons. First, the presence of ctDNA after tumor resection has been shown to indicate evidence of residual disease and hence points to high risk of recurrence in patients with early breast cancer receiving neoadjuvant chemotherapy,^82^ with stage II colon cancer,^83^ and with non-small-cell lung cancer (NSCLC).^73^ Hence, ctDNA testing may be capable of identifying patients in need of very close monitoring. Second, detailed characterization of the resected primary tumor allows the design of patient-specific assay panels and the development of personalized markers. To this end, taking leverage of somatic structural rearrangements, e.g., translocation breakpoints, yields biomarkers with high sensitivity and specificity, as these aberrant fusions of DNA sequences do not occur in non-tumor cells.^84–87^ Another option is the aforementioned barcoding strategy combined with the simultaneous testing of a large number of mutations and the use of a threshold for a minimum number of mutations needed to be detected for a plasma sample to be called tumor-positive.^73,78,81^ These strategies have been shown to detect relapse with lead time compared to standard techniques. However, a present drawback is that no clinical guidelines for handling plasma DNA-based information exist yet. For example, breakpoint mutant DNA molecules may be detected at levels as low as 0.001%.^84^ If a follow-up analysis reveals an increase of such a patient-specific marker from 0.001% to—let’s say—0.05% in the plasma DNA, but standard imaging does not show evidence for relapse, there are no guidelines whether and how this should affect the clinical management of the respective patients. Furthermore, the early detection and characterization of potential disease recurrence at the genomic level would help stratify patients who might benefit from a particular adjuvant regimen and would provide a platform for tracking adjuvant treatment response. For example, in patients with stage II colon cancer treated with adjuvant chemotherapy, the persistent presence of ctDNA after therapy indicated a reduced recurrence-free survival.^83^ Similarly, it was reported that in NSCLC patients in-depth characterization of postoperative plasma allows identification of adjuvant chemotherapy resistance and hence patients with a high likelihood for disease recurrence.^73^

The second scenario includes screening of at-risk persons (Fig. 3b), i.e., due to a hereditary predisposition, such as germline mutations in *BRCA1* or *BRCA2*, or in a mismatch-repair gene predisposing to Lynch syndrome, or because of chronic exposure to toxic agents, e.g., smokers with their increased risk for lung cancer, or due to viral infections. In any case, due to the known risk pattern, pre-knowledge exists which can be leveraged to tailor screening tests for specific organs. In such cases, sensitivity can be improved by extending analyses beyond blood samples by additionally sampling other body fluids or cytological specimen which are close to the organ at risk. Pioneering studies have already shown several decades ago that tumor-specific mutations can be identified in urine of patients with bladder cancer^88^ and in the stool of patients with curable CRC.^89^ This strategy, which has also been referred to as “proximal sampling”,^16^ has recently been extensively employed for the detection of mutations associated with ovarian cancer in ovarian cyst fluids,^90^ from lavage of the uterine cavity,^91^ from tampons,^92^ or from Papanicolaou (Pap) smears.^93^ Other examples include the analysis of saliva for the detection of head and neck squamous cell carcinomas^94^ or cerebrospinal fluid to obtain information about brain and spinal cord tumors.^95,96^ Hence, pre-knowledge about endangered organs significantly extends options for analyses which may significantly facilitate early detection efforts. Regarding cancer entities associated with viral infections, a recent study reported that screening for Epstein-Barr virus (EBV) DNA in plasma allows the detection of early asymptomatic nasopharyngeal carcinoma.^97^ A high specificity was achieved by focusing on a target sequence, which is repeated about ten times within the EBV genome. As each nasopharyngeal tumor cell contains approximately 50 EBV genome copies, the target sequence is actually present about 500 times in each cell, which greatly facilities its detection when released into the circulation. Furthermore, to distinguish EBV signals from infections, the investigators repeated the evaluation after 4 weeks to identify those individuals with persistent positive results.^97^ Conducting this study in an endemic area, a positive predictive value (PPV) of 11% was achieved; however, this PPV may drop if such a screening is conducted outside of endemic areas or high-risk persons.^97,98^ Nevertheless, this study illustrates that plasma DNA screening may allow early detection of cancers associated with viral infections.

The third scenario is the toughest group, i.e., the general population, where no pre-knowledge about the individual risk or organs at risk exists, i.e., there is no clue as to where to search and what to look for (Fig. 3c). Due to the lack of tumor material, patient-specific assays cannot be designed for screening and proximal sampling strategies will likely be insufficient without any knowledge regarding at-risk organs. Furthermore, as outlined above, phenomena such as the occurrence of mutations in driver genes in non-tumorous tissue and clonal expansions will likely be reasons as to why approaches based on mutation screening alone will be insufficient. Hence, it is likely that more robust results can be achieved by other liquid biopsy strategies.

In addition to these three main early detection scenarios, the early detection of resistant clones is clinically of utmost importance. Although targeted therapies have expanded upon cancer treatment options over the last decade, the tumor heterogeneity and complexity of clonal evolution and selection discussed previously almost inevitably lead to the development of resistance to systemic treatment in a large number of tumors. Various landmark studies have already exemplified the use of ctDNA to make temporal measurements of total tumor burden and to identify the presence of mutations conferring resistance to therapy with considerable lead time compared to routinely used imaging procedures.^31,42,74,99–102^ Not only can this approach help detect prevalent mutations with known associations of acquired resistance, but it can also indicate the presence of novel associated mutations and offer new insight into their role in the buildup to resistance, as was demonstrated by the development of *NRAS* codon 61 mutations in 62.5% of profiled CRC patients progressing after EGFR blockade.^42^ Since newly detected clones in the circulation may derive from minor pre-existing clones originating in the primary tumor^74^ as well as from ongoing mutagenesis, it has been suggested that multiple resistance mechanisms may exist simultaneously, thus highlighting the need to monitor the change in clonal burden with time.^102^

## Future liquid biopsy extensions

As mentioned above, more robust results can likely be achieved, in particular for 3rd scenario cases, by extended liquid biopsy strategies involving more parameters. Such alternative strategies include the extraction of additional information from plasma DNA and furthermore by combining analyses of various other components. Additional information from plasma DNA can be obtained by bisulfite sequencing and methylation deconvolution of the sequencing data, which may provide information about the tissue of origin of the plasma DNA.^103–105^ Furthermore, as plasma DNA is nucleosome-protected DNA, nucleosome-position maps can be directly established from cfDNA, which can also inform about the tissue of origin^106^ or even provide information about the expression status of genes from the cells which released their DNA into the circulation.^107^

An intensely debated issue is the plasma DNA fragment size,^108^ as recent studies have demonstrated that ctDNA is shorter than cfDNA from non-tumor cells.^109,110^ Therefore, there are extensive efforts to increase the proportion of smaller (<100 bp) cfDNA fragments either by different DNA library preparation protocols^106,111^ or excision of DNA with appropriate size patterns from polyacrylamide gels.^110^ Whether these efforts result in an enrichment of a specific plasma DNA subpopulation and may hence increase resolution is a matter of debate at present.^110,112,113^

However, options for combination strategies appear to be almost endless, as peripheral blood contains a number of other components released by cells into the circulation, such as mRNA, microRNA, extracellular vesicles, proteins or cancer metabolites. Since several of these additional components released into the circulation have gained much recent attention regarding their clinical utility for supplementing the liquid biopsy, they will be addressed here briefly.

The “circulating transcriptome”, which encompasses miRNAs, lncRNAs and mRNAs, could provide additional clinical value in assessing tumor-specific changes. The presence of circulating tumor-derived mRNA may allow the identification of tumor-specific gene expression profiles (reviewed in refs. 17, 21, 114). However, extracellular mRNA proves difficult to detect in the circulation, as the majority of these molecules are promptly degraded by RNase activity. In contrast, miRNAs demonstrate remarkable stability in blood and are also known to circulate in the protected form of exosomes, which are microvesicles carrying functional biomolecules that can be transferred horizontally to recipient cells.^115,116^ Furthermore, tumor-educated platelets were recently identified as another source for miRNAs.^117,118^ As miRNA signatures in blood appear to reflect the miRNA pattern from the corresponding tumor,^119^ it is likely that miRNAs will remain at the forefront of biomarker research due to their accessibility and diagnostic potential. Furthermore, lncRNAs, another class of noncoding RNAs which are greater than 200 nucleotides in length, are also stable in blood, as they are protected from endogenous RNases. Although they have not been studied as extensively as miRNAs, circulating lncRNAs may evolve to noninvasive biomarkers for tumor diagnosis.^120^

The potential of proteomic applications for the early detection of cancer has been explored for a long time,^121^ but many protein-based tests have not reached the sensitivity and specificity needed in clinics. Recent advances in proteomics and peptidomics have provided novel means for adding such molecules to the arsenal of the liquid biopsy approach. Some studies utilized exosomes, as they contain not only nucleic acids but also proteins and some of these proteins may be exclusively present in cancer exosomes. An example is the membrane-anchored protein Gypican-1, whose occurrence in circulating exosomes was reported to allow the detection of early stages of pancreatic tumors.^122^ In addition to exosome-related proteins, blood-based protein tests were developed for a variety of tumor entities, e.g., for prostate cancer detection, combinations of total and free prostate-specific antigen levels with other biomarkers were applied.^123^ Furthermore, protein analyses were applied to other body fluids such as urine to discriminate between healthy individuals and individuals with renal cell carcinoma with high specificity and sensitivity^124^ or to distinguish urothelial carcinoma patients with non-muscle and muscle-invasive subtypes.^125^

From such new developments, novel multi-marker panel assays may evolve with suitable sensitivity and specificity for clinical application. Hence, it is very likely that future liquid biopsy strategies aiming at early detection will encompass a multitude of different parameters and that such multiparametric analyses may change current views about resolution limits.

## Conclusions

We outline here the enormous biological and technical challenges which liquid biopsies have to meet to detect precursor lesions or early cancer stages. At present, there is still no general concept describing an approach for early detection of cancer with liquid biopsies, but multiple unexplored options remain to be tested. Critical questions that must be addressed to advance the field of liquid biopsies applied toward “earlier” detection are summarized in Table 1. Aside from outstanding technical issues, one of the most important biological issues remains to be the currently unknown biology of ctDNA release, and in terms of clinical applications—due to the lack of experience and appropriate studies—the missing clinical guidelines. Importantly, we may have to adjust our perception regarding the success of early cancer detection strategies. For the reasons outlined in this review, it is unlikely that a liquid biopsy-based test will ever achieve early detection with a 100% specificity and sensitivity and there will probably be no test capable of detecting all tumor entities. However, a test which may enable early detection to a certain percentage for a subset of cancers could already have a great impact on the life of many individuals.

### Data availability

Data sharing not applicable to this article as no datasets were generated.
